# Analysis of stress distribution in ceramic and titanium implants in alveolar sockets of the anterior region of the maxilla

**DOI:** 10.4317/jced.55945

**Published:** 2019-10-01

**Authors:** Cacilda-Cunha Ferraz, Rosália-Moreira Barros, Fábio-Cunha Ferraz, Átila-Augusto Mundstock, Bruno-Sotto Maior

**Affiliations:** 1 Department of Dentistry, São Leopoldo Mandic Dental Research Center, rua Rua Marechal Floriano 600/1009, Centro, Governador Valadares/MG, Campinas - São Paulo -Brazil

## Abstract

**Background:**

In the routine of dentistry, knowing the biomechanical properties of implant systems and their inherent stress distribution under force loading is an essential step to predict structural damage and biological responses. This study aimed to investigate stress distribution in zirconia and titanium implants and their biomechanical response in alveolar sockets of the anterior region of the maxilla through tridimensional finite element analysis.

**Material and Methods:**

From computed tomography scans of a reference patient, three models of the maxillary dental arch were designed with Rhinoceros 5.0 software (McNeel Europe™, Barcelona, Spain). In each model, a dental implant replaced the maxillary left central incisor. The implants consisted of M1) Zirconia Pure Ceramic Implant Monotype; M2) Zirconia Pure Ceramic ZLA; and M3) Titanium Bone Level - Roxolid SLA. Ceramic crowns were installed in all the implants. Implants and prostheses were loaded with 50N oblique and axial forces. Von-Mises and Mohr Coulomb criteria were used to assess stress distribution in the implant systems and perimplantar bone, respectively.

**Results:**

Traction was detected in the cervical region of the palatal bone surface of all the models. Oppositely, compression was found in the cervical region of the vestibular bone surfaces.

**Conclusions:**

Zirconia Pure Ceramic Implant Monotype had the best response under oblique force loading. Ceramic implants may be an alternative to replace titanium implants in fresh alveolar sockets in the anterior region of the maxilla.

** Key words:**Finite elements, implants, stress, ceramic, titanium.

## Introduction

Oral Implantology experienced evident improvements since the development of osseointegrated implants (1). Design, materials and surgical techniques gradually evolved to enable optimal clinical performances (2). Currently, titanium implants represent the most common choice for oral rehabilitation because of inherent biological compatibility and biomechanical properties (3).

In the routine of dentistry, challenging situations, such as allergy to metal and the need for aesthetic outcomes, may require alternative tools (4). In particular, thin gingival phenotypes, recession, bone loss, and the dark color of titanium implants conflict with the interest of patients that seek for aesthetic solutions (3). Zirconia recently emerged as a proper alternative (3). The advantages of these implants extend to fracture resistance and, eventually, less accumulation of biofilm compared to titanium (3,5-7).

Despite the improvements in Oral Implantology, scientific knowledge of implants and their contribution to oral rehabilitation must be progressively encouraged to promote evidence-based practices (8,9). Studies in stress distribution figure amongst those with direct contribution to practice. Finite element analysis combined with applied force loading allows a qualitative and quantitative assessment of stress distribution on the internal and external implant surfaces (10).

Considering the fact that I) zirconia implants represent an innovative contemporary trend in Oral Implantology, II) finite element analysis is an advanced approach to assess stress distribution, and III) immediate implant placement is a promising technique to reduce the number of surgical interventions and preserve the adjacent bone support (11-13), this study aimed to investigate by means of finite element analysis stress distribution in zirconia and titanium implants placed in fresh alveolar sockets.

## Material and Methods

-Study design and ethical aspects

This analytical study was performed with institutional ethical clearance.

-Study set up

From a reference patient, computed tomography scans were retrospectively obtained for dental treatment purposes. The scans were used for creating (Rhinoceros 5.0, McNeel Europe™, Barcelona, Spain) and processing (Ansys Workbench® 16.0, Canonsburg, PA, USA) three-dimensional models of the maxillary dental arch. The models were designed with a thin (nearly 1mm) layer of cortical bone and medullar bone. Three models were obtained for placing implants in the region of the maxillary left central incisor (tooth #21). The implants (Institute Straumann AG™, Basel, Switzerland) measured 4.1x14mm and consisted of M1) Zirconia Pure Ceramic Implant Monotype; M2) Zirconia Pure Ceramic ZLA; and M3) Titanium Bone Level - Roxolid SLA. CI RD Straumann 5.5mm PURE base and RC Variobase 5.5mm abutments were attached to the Zirconia Pure Ceramic ZLA and Titanium Bone Level implants with their inherent screws, respectively. IPS E-max press ceramic crowns (Ivoclar Vivadent AG™, Shaan, Liechtenstein) were installed over all the implants.

-Data collection

Once the implants were placed, measurements were taken from the cortical and medullar bone, implants, abutments, screws and prostheses before and after force loading ( Table 1). The scientific literature (14-16) was consulted to set up references for the mechanical properties of the model parts used in this study. Implants were considered non-osseointegrated when they presented frictionless contact with the adjacent bone. Frictional non-linear contacts were simulated with a coefficient of 0.41 for contact between titanium structures and 0.25 between zirconia and titanium (17). Other simulations were performed without sliding and promoting gap formation.

**Table 1:**
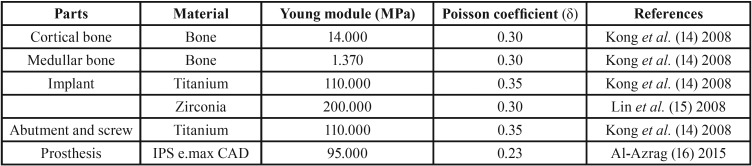
Mechanical properties of the model parts used in this study.

-Data analysis

Finite element models were built with tetrahedral elements that ranged in size from 0.1-0.5mm. The number of elements varied between 583.656 and 615.095, and the number of joint vertices between 861.042 and 916.195. Digitally, the models were fixed on their laterals in the region of cortical and medullar bones.

In each model, axial and oblique forces of 50N were applied. In the former, force was loaded in the cingulum parallel to the vertical axis of the implant. In the latter, force was loaded within 45° on the palatal surface of the crown close to the incisal edge. A computer-guided color map was generated after force loading. The analysis was three-dimensional, linearly elastic, homogeneous, isotropic and with plane deformation state to simplify the method. Von-Mises (Mpa) maximum and minimum values were used to assess stress distribution in static elements, such as implants and prosthetic components. The Mohr-Coulomb criterion was used to quantify structural damage risk to the adjacent bone. In particular, it considers the difference in the impact of traction and compression stresses on friable material (such as bone) and the consequent impact on the generation of bone damage.

## Results

**Figure 1:**
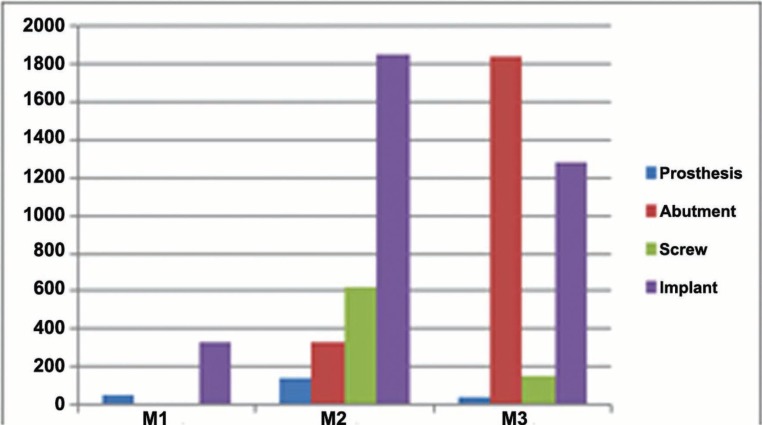
Von-Mises (MPa) stress distribution under oblique force loading.

**Figure 2:**
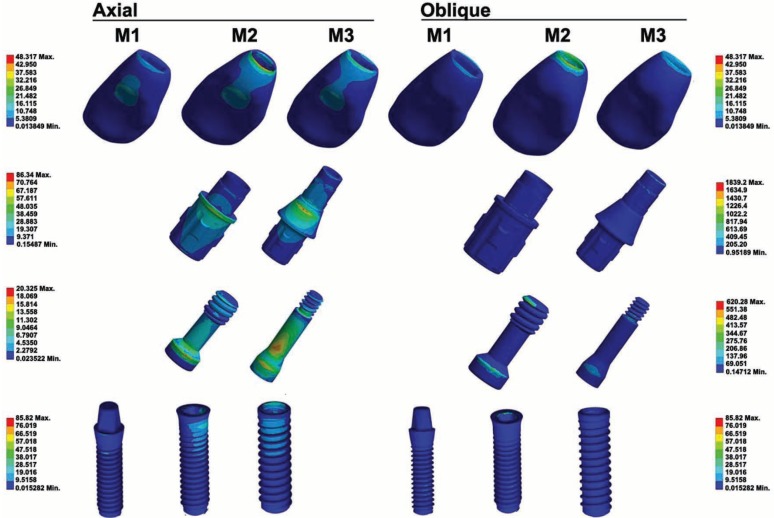
Color map of stress distribution on the prosthetic crown, abutment, screw and implant of samples M1 (Zirconia Pure Ceramic Implant Monotype), M2 (Zirconia Pure Ceramic ZLA) and M3 (Titanium Bone Level - Roxolid SLA).

Under oblique forces, stress distribution was more concentrated in the Zirconia Pure Ceramic ZLA implant and in the abutment of the Titanium Bone Level - Roxolid SLA implant. Zirconia Pure Ceramic Implant Monotype presented the lower levels of stress. In relation to the screw, Zirconia Pure Ceramic ZLA presented higher stress than the others (Fig. 1). In Zirconia Pure Ceramic Implant Monotype and Zirconia Pure Ceramic ZLA, the applied force overloaded the implant, while in Titanium Bone Level - Roxolid SLA system the stress concentrated more in the abutment. In particular, the stress in the abutment of the latter was considerably higher than the other systems. In the prosthetic crown, stress was more evident in Zirconia Pure Ceramic ZLA system. When the applied forces changed from axial to oblique, stress was higher in Zirconia Pure Ceramic ZLA. In this context, the lower stress values were observed in Zirconia Pure Ceramic Implant Monotype (Fig. 2).

-Comparative outcomes for bone responses (traction x compression)

**Figure 3:**
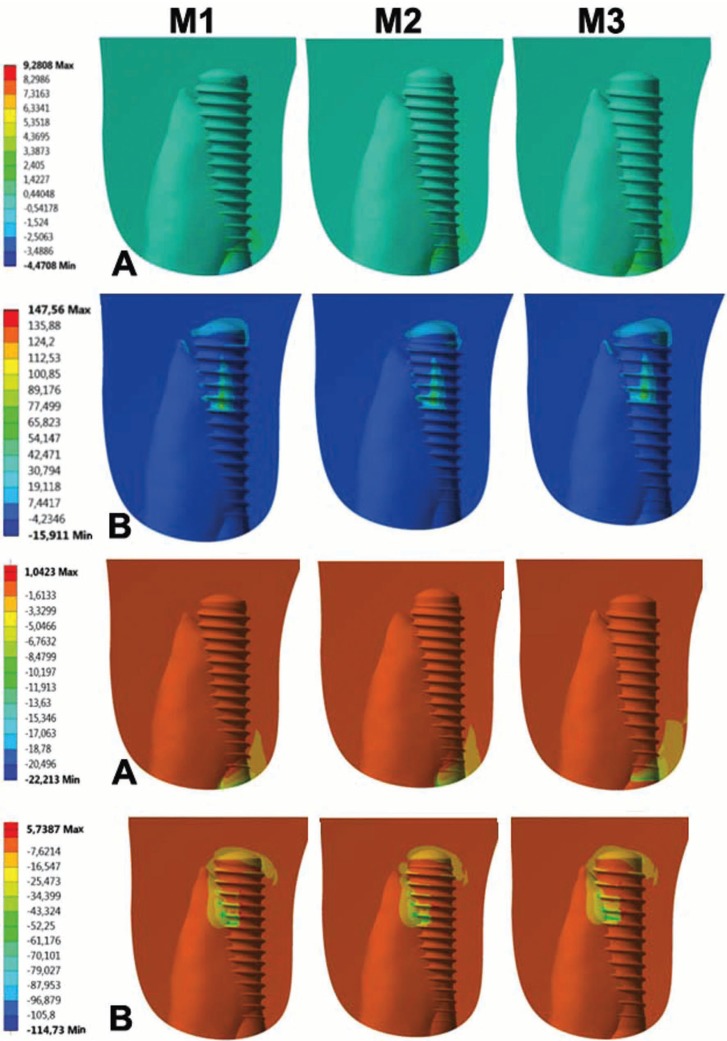
Color map of stress distribution on the adjacent bone after axial (A) and oblique (B) in samples M1 (Zirconia Pure Ceramic Implant Monotype), M2 (Zirconia Pure Ceramic ZLA) and M3 (Titanium Bone Level - Roxolid SLA).

Under axial force, the higher (9.2808 Mpa) and lower (57408 Mpa) stress values were observed in Zirconia Pure Ceramic ZLA and Zirconia Pure Ceramic Implant Monotype, respectively ( Table 2; Fig. 3). Under oblique force, the higher (147.56 Mpa) and lower (113.15 Mpa) stress values were observed in Titanium Bone Level - Roxolid SLA and Zirconia Pure Ceramic ZLA, respectively ( Table 2; Fig. 3). Proportionally, the highest difference between systems was found comparing Titanium Bone Level - Roxolid SLA and Zirconia Pure Ceramic ZLA. The former expressed 30% more stress (traction) under oblique force ( Table 2).

**Table 2:**
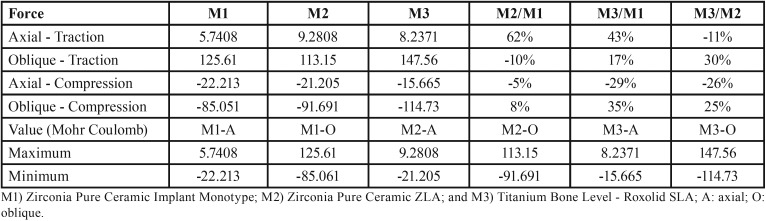
Comparison of stress distribution (traction and compression) in bone and expression of stress distribution under axial and oblique forces according to Mohr Coulomb criteria.

Under axial force, compression was higher in Zirconia Pure Ceramic Implant Monotype (22.213 Mpa). Zirconia Pure Ceramic ZLA had similar outcomes (21.205 Mpa). Titanium Bone Level - Roxolid SLA showed the lowest values (15.665 Mpa). On the other hand, the latter showed the highest (114.73 Mpa) stress distribution under oblique force. Lower and similar values were found in Zirconia Pure Ceramic Implant Monotype and Zirconia Pure Ceramic ZLA (the proportional difference between both ranged below 10%).

Higher risk of bone damage was found under oblique force loading. However, all the Mohr Coulomb values were below 1 – indicating lack of bone damage by rupture ( Table 2).

## Discussion

The scientific literature on the biomechanical response of implants in fresh alveolar sockets increased with studies that investigated the indication of zirconia implants to patients allergic to metal. This study was designed to assess the biomechanical response of ceramic and metallic implants in fresh alveolar sockets in the anterior region of the maxilla.

Finite element analysis was used in the methodological set up. In science, this method enables bi- and three-dimensional realistic simulation of the masticatory load over implants and prosthetic components. Additionally, it allows a deeper visualization of the interaction between implant and bone. Consequently, it represents a useful tool to understand stress and deformation and predict eventual damage to the adjacent cortical and medullar bone. The quantified data obtained within finite element analysis shows evidence not commonly found in experimental studies (18).

The importance of investigating the interaction of implant and bone from a biomechanical point of view relies on the structural reorganization (formation, resorption or maintenance) of the bone triggered by stress and deformation. Clinically, resorption may be translated into implant loss (18). Assessing bone-implant interaction focusing in stress and deformation is not feasible with clinical studies.

Other methodological aspects to be considered involve the assessment of Von-Mises criteria and the investigation of oblique force loading. Von-Mises criteria were established within the methods because they are used in the scientific literature to study stress in fragile materials, such as bone (19). Oblique force loading was alternatively implemented to axial loading because it represents the most harmful force to the implant system (20).

-Implants

After axial and oblique force loading, an increase in Von-Mises stress distribution was observed in the implant systems. In implant system M3, the stress was concentrated in the abutment. These outcomes suggest that ceramic implants (one-piece or not) absorb more stress due to their harder material (zirconia) and different geometry. It also must be noted that zirconia implants are placed above the bone level, while titanium implants are inserted up to the bone level – the difference changes the rotation center of ceramic implants and concentrates more stress in the screw and abutment.

Qualitatively, all the implants presented stress in the cervical region of the vestibular surface. These outcomes are explained by the oblique force applied over the abutment and by the sharp-edged region under pressure that propitiates the concentration of stress. Quantitatively, all the implants presented deformation.

Comparisons between implants showed that systems M2 and M3 presented more stress than system M1. From a bio-mechanical point of view, zirconia implants were more beneficial to the bone because they presented more tension and less compression stress. Accordingly, system M3 is less indicated for fresh alveolar sockets because of the higher compression that hampers osseointegration. Osseointegration is a key factor to assure success in rehabilitation with implants. Avoiding marginal bone loss also figures with evident importance. In this context, single-piece zirconia implants reveal similar or better performance than natural teeth.

From a qualitative assessment, all the risk factors must be considered. Biologically, the cortical bone must be stronger than the medullar bone. This structural set up promotes a better stability of the implant and distribution of stress. In other words, it decreases the risk factor of loosing implant system M3. However, positioning the implant at bone level possibly increased the risk because the bone region under stress (cervical region) is not robust and unable to optimal stress distribution (21).

Based on the exposed, implant system M1 had a lower risk of bone loss compared to implants M2 and M3. Specifically, because system M1 was set up with a single body, it had lower stress compared to multiple-part systems. In addition, all the samples showed higher stress in the cervical region of the implants – corroborating previous studies (22-26).

Quantified outcomes show that higher stress was found in system M2, while lower values were found in system M1. The same trend maintained when force changed to oblique. In particular, the transition to oblique force overloaded the implant in systems M1 and M2. In system M3, the implant also was affected, but the abutment showed more quantified stress values.

-Abutments, screws and prostheses

The outcomes of this study were proportionally considered based on the flow-limit (maximum stress limit before plastic deformation). Screws also were included in the analysis and they showed inherent accumulated stress even before the force loading.

In the prostheses, system M2 absorbed more stress under oblique force than systems M1 and M3. The screw of system M3 presented more significant differences than system M2. The differences between systems may be explained by their mechanical resistance, materials and geometry. Possibly, system M1 showed less stress because of its solid single-piece body. Differently, implants with two pieces (i.e. implant and abutment) are more fragile.

The highest stress values were found between the head and the body of the screws, which represent the contact interface between screw and implant and between implant and abutment. Consequently, the applied force is distributed throughout screw and abutment inside the implant (5,27) confirm this phenomenon by showing that oblique forces lead to high stress in implant components, prosthetic crowns and cortical bone.

In relation to the abutment, system M3 showed higher stress compared to M2. It is justified by the fact that oblique forces project the abutment towards the vestibular direction. Simultaneously, the implant resists in the lower region flexing the abutment and adjacent components. Additionally, the implants protect the abutment below the platform. However, the region above the platform is not reinforced and is more susceptible to accumulation of stress. The peak of stress observed in this study confirms the fact that the resistance is provided by the abutment.

The analysis of the screws showed higher stress in system M2 compared to M3. The rationale behind the difference may be explained by the participation of titanium and zirconia in the process of stress distribution. Clinically, the lower stress detected in the screw of system M3 potentially decreases loosening over the time. Similar outcomes were found in the literature (28) during the comparison of titanium and zirconia systems. In specific, two-piece screwed zirconia systems had higher failure rates compared to titanium systems (28). Failure concentrated in the interface between abutment and implant and around the screw – highlighting that connection systems must be optimally designed to avoid failure.

-Perimplantar bone 

Bone remodeling may be induced by applied stimuli (15). However, the exact mechanism that mediates this process remains uncertain.

In this context, Mohr-Coulomb criteria were used to quantify, in structural level, risk to damage. This approach was chosen because it considers the different impacts of stress (traction and compression) and damage in the bone (29).

Specifically, this study showed that stress concentrated in the level of alveolar bone crest. Moreover, all the samples showed concentrated stress in the vestibular region of the implant system. However, it is important to note that the simulations performed in the present study were static. In the clinical practice other forces may occur. Hence, future investigations with different set ups are necessary to clarify these outcomes.

Qualitatively, all the samples revealed stress in the cervical vestibular surface of the bone crest in contact with the implant. This phenomenon is justified by the direction of the oblique force that compresses the implant against the internal wall of the alveolar socket. Despite the predominance of compressive stress in the region, the inherent bone deformation leads to a peripheral traction that increases the risk of damage to the interface of bone-implant (30). As expected, stress distribution in axial forces was lower than oblique forces. While in axial force loading stress was more distributed in the cervical region of the vestibular surface, in oblique loading it concentrated in the central and apical regions of the vestibular surface – region of anchorage of implants in fresh alveolar sockets.

The methodological decision for studying implant with Cone Morse connection was founded on the fact that these implants are broadly used in the clinical practice. The outcomes presented in this study corroborate the available literature (31). These implants have the advantage of precise fitting and lack of microgaps between implant and prosthesis – decreasing the risk of bacterial infection (31). Additionally, Cone Morse systems also induce a better transmission of force from the abutment to the implant culminating in an optimal distribution of stress throughout the internal walls of the implant towards the bone. This process protects the screw and the abutment and results in a stable unit that enables proper osseointegration in fresh alveolar sockets (32).

The force addressed in this study (50N) is related to the mechanical stability of the implant in the fresh socket. Ideally, deleterious spots of stress must be absent from the bone-implant interface under this amount of force. During masticatory activity, the unit that consists of implant and prosthesis must survive stress and enable osseointegration by distributing it through the prosthetic crown, cement, screw, abutment, implant and bone (33).

The specific criteria that guarantee success in oral rehabilitation with implants are constantly discussed. However, achieving and maintaining osseointegration and avoiding marginal bone loss are consolidated cornerstones to be considered (34).

The scientific literature shows that implant placement in fresh alveolar sockets (without preventive measures) may lead to structural bone changes, such as reduction in the length of the bone crest and palatal displacement (35). Clinically, these changes may culminate in aesthetic impairment. Oppositely, proper bone height and thickness must be preserved to guarantee a harmonious gingival contour around the implant (35).

The validation of this study requires in vivo investigations of zirconia and titanium implant placement in fresh alveolar sockets of the anterior region of the maxilla.
